# Psychische Gesundheit Studierender während des Online-Studiums im Zuge der COVID-19-Pandemie – quantitative und qualitative Befunde

**DOI:** 10.1007/s11553-023-01046-3

**Published:** 2023-05-26

**Authors:** Lisa Giesselbach, Janna Leimann, Carolin Bonner, Jan Josupeit, Sven Dieterich, Eike Quilling

**Affiliations:** Department für Angewandte Gesundheitswissenschaften, Hochschule für Gesundheit Bochum, 44801 Bochum, Deutschland

**Keywords:** Studentisches Gesundheitsmanagement, Online Studium, Wohlbefinden, Depressionen, Studierendengesundheit, Student health management, Online studies, Well-being, Depressions, Student health

## Abstract

**Hintergrund:**

Studierende gelten auch in der COVID-19-Pandemie („coronavirus disease 2019“) als besonders vulnerabel für psychische Belastungen und Erkrankungen.

**Ziele:**

Im Rahmen der vorliegenden Studie wurde eine quantitative Bestandsaufnahme der psychischen Gesundheit Studierender an der Hochschule für Gesundheit in Bochum vorgenommen. Darüber hinaus wurde im Zuge einer qualitativen Untersuchung ihr subjektives Erleben des Online-Studiums im Wintersemester 2020/2021 erhoben.

**Methoden:**

Im Rahmen einer online-basierten Umfrage wurden im März 2021 mittels validierter Instrumente das Wohlbefinden (WHO-5) und das mögliche Vorliegen depressiver Störungen (PHQ-9) bei 435 Studierenden der Hochschule für Gesundheit in Bochum erhoben. Weitergehend wurden Studierende der Hochschule zu ihren Erfahrungen und ihrem Erleben im Online-Studium interviewt.

**Ergebnisse und Schlussfolgerung:**

Es zeigte sich im Vergleich zur Allgemeinbevölkerung, dass das Wohlbefinden der Befragten unterdurchschnittlich ausgeprägt war und dass mit 26,9 % bei überdurchschnittlich vielen Befragten begründeter Verdacht hinsichtlich des Vorliegens von Depressionen bestand. Die Ergebnisse aus dem qualitativen Forschungsstrang bildeten ein überwiegend negativ bewertetes Erleben der Studierenden bezüglich des Online-Studiums ab. Insbesondere fehlende soziale Kontakte sowie Schwierigkeiten in der digitalen Umsetzung von Lehrveranstaltungen standen hiermit im Zusammenhang. Die Daten deuteten v. a. auf die erhebliche Bedeutung von Hochschulen und des Studiums in Bezug auf die psychische Gesundheit von Studierenden. So besteht v. a. dahingehend Handlungsbedarf, sich als wichtiger Akteur hinsichtlich des Auslösens von psychischer Beanspruchung der Studierenden einerseits sowie als Ort gesundheitsförderlicher Interventionen andererseits zu verstehen. Die Betrachtung beider Perspektiven bietet diverse Ansatzpunkte für Gesundheitsförderung bzw. -management in Hochschulen.

## Abstract (en)

**Background:**

Students are also considered particularly vulnerable to mental stress and illness during the coronavirus disease 2019 (COVID-19) pandemic.

**Objectives:**

In the present study, a quantitative inventory of the mental health of students at the University of Applied Sciences (“Hochschule für Gesundheit Bochum”) was conducted. In addition, their subjective experience of online classes in the winter semester 2020/2021 was surveyed in the course of a qualitative study.

**Methods:**

Within the framework of an online survey, well-being (WHO-5) and possible presence of depressive disorders (PHQ-9) of 435 students at the “Hochschule für Gesundheit Bochum” (Germany) were surveyed in March 2021 using validated instruments. Furthermore, students of the university were interviewed about their experiences with online courses.

**Results and conclusion:**

Compared to the general population, the well-being of the respondents was below average and an above average percentage of respondents (26.9%) had a reasonable suspicion of depression. The results from the qualitative research showed a predominantly negative assessment of the students’ experience with regard to online courses. In particular, a lack of social contacts and difficulties in the digital implementation of courses gave rise to this impression. Above all, the data pointed out the considerable importance of universities and studying with regard to the mental health of students. Thus, there is a need for action, especially in terms of understanding oneself as an important actor with regard to triggering students’ mental stress, on the one hand, and as a place for health-promoting interventions, on the other. Consideration of both perspectives offers various starting points for health promotion and health management in universities.

## Hintergrund und Fragestellung

Am 11. März 2020 wurde die globale Verbreitung des Coronavirus SARS-CoV‑2 („severe acute respiratory syndrome coronavirus 2“) von der Weltgesundheitsorganisation (WHO) zur weltweiten Pandemie, zur sog. COVID-19-Pandemie („coronavirus disease 2019“), erklärt [31]. Die Pandemie und die in der Folge ergriffenen Maßnahmen zum Infektionsschutz beeinflussten den Alltag und das Leben von Menschen erheblich und bergen multidimensionale Stressoren auf verschiedenen Ebenen u. a. für die psychische Gesundheit [5].

Diese wird von der WHO [30] als „Zustand des Wohlbefindens beschrieben, in dem der Einzelne seine Fähigkeiten ausschöpfen, die normalen Lebensbelastungen bewältigen, produktiv und fruchtbar arbeiten kann und imstande ist, etwas zu seiner Gemeinschaft beizutragen“. Der Begriff Wohlbefinden („well-being“) wird in der modernen psychologischen Forschung mit Glück gleichgesetzt und geht mit einer hohen subjektiven Lebenszufriedenheit sowie häufig positiver und selten negativer Stimmung einher [8]. Es zeigt sich daher, dass das Verständnis von psychischer Gesundheit nach den Definitionen der WHO stark von dem subjektiven Empfinden und den Lebensumständen der Betroffenen abhängt. Aus diesem Grund ist neben der Betrachtung des Gesundheitszustands die Betrachtung der Lebensbedingungen für eine ganzheitliche Betrachtung der psychischen Gesundheit unabdingbar.

### Anstieg von Angstsymptomen und Depressionen im Rahmen der COVID-19-Pandemie

Insbesondere aufgrund der unterschiedlichen Risiken, die Personen mit bestimmten Merkmalsausprägungen und Lebenssituationen in der COVID-19-Pandemie im Hinblick auf die psychische Gesundheit haben, spielt auch die Betrachtung spezifischer Gruppen eine besondere Rolle. Zu Beginn der Pandemie 2020 zeigen Reviews internationaler Studien einen generellen Anstieg von Angstsymptomen, Depressionen und Stress auf [26, 32]. Insbesondere Menschen mit psychischen Vorerkrankungen und im Gesundheitswesen arbeitende Personen wurden als vulnerabel für psychische Erkrankungen identifiziert [28]. Untersuchungen sozioökonomischer Folgen der Verbreitung des Coronavirus in Deutschland, kurz SOEP-CoV-Studie konnten im April 2020 einen Anstieg der Depressions- und Angstsymptomatik im Vergleich zum Vorjahr abbilden, diesen bezeichnen die Autoren und Autorinnen jedoch als nicht außergewöhnlich hoch [9]. Im Rahmen der SOEP-CoV-Studie wurden zwei Erhebungen durchgeführt. Die zweite Erhebung im Februar 2021 konnte einen Anstieg von Angst- und Depressionssymptomen bei einer Verschlechterung des allgemeinen Wohlbefindens und der Lebenszufriedenheit aufzeigen [10]. Hier wurden speziell junge Menschen und Frauen als Risikogruppe für psychische Erkrankungen während der COVID-19-Pandemie identifiziert.

Psychische Erkrankungen wie Depressionen stellen mit einer deutschlandweiten Prävalenz von 9,2 % eines der größten Gesundheitsrisiken der aktuellen Zeit dar. Auch hier sind Jüngere mit 11,5 % deutlich häufiger betroffen als ältere Menschen mit 5,2 % [17]. Depressionen stehen in direktem Zusammenhang mit erheblichen individuellen als auch gesamtgesellschaftlichen Belastungen. Die Anzahl der Krankentage aufgrund psychischer Erkrankungen ist in den letzten 12 Jahren um 53,2 % gestiegen. Im Jahr 2021 gab es zudem mehr Fälle aufgrund psychischer Erkrankungen als aufgrund von Herz-Kreislauf-Erkrankungen [23]. Um diesem Trend entgegenzuwirken, sind die Förderung der psychischen Gesundheit und die Prävention psychischer Erkrankungen besonders relevant.

### Studierende sind in hohem Maße psychisch belastet

Eine Personengruppe, die bereits vor Ausbruch der Pandemie überdurchschnittlich häufig psychische Belastungen im Vergleich zur körperlichen Gesundheit und der Allgemeinbevölkerung zeigten, sind Studierende [25]. Eine besondere Vulnerabilität kann bei ihnen aufgrund unsicherer, sich verändernder und teils prekärer Lebensumstände vermutet werden, die darüber hinaus auch im Kontext der sog. Transitionsphase, in der sich junge Erwachsene im Übergang vom Kindes- ins Erwachsenenalter befinden, beschrieben ist [21,4]. So konnte eine deutschlandweite Untersuchung Studierende vor Ausbruch der Pandemie insbesondere im Bereich der psychischen Gesundheit als besonders belastet identifizieren [16].

Untersuchungen, welche die psychische Gesundheit Studierender während der COVID-19-Pandemie untersuchen, zeigen Hinweise auf eine Verschlechterung der psychischen Gesundheit [3, 11].

Bisherige Befragungen von Studierenden zeichnen ein überwiegend negativ geprägtes Bild in Bezug auf das pandemiebedingte Online-Studium. Im Rahmen der bundesweiten Studienreihe Studieren in der Pandemie (Studi Co I und II) wurde die Studierendenperspektive auf die Coronapandemie im Juli 2020 und 2021 untersucht. Die digitale Arbeitssituation wird von den Studierenden zum zweiten Erhebungszeitraum besser eingeschätzt als zu Beginn der Pandemie. In beiden Semestern werden jedoch das Fehlen sozialer Kontakte und ein mangelnder Austausch im Hochschulsetting kritisiert. Vorteile werden in dem Wegfall von Fahrtwegen und erhöhter Flexibilität in der Arbeitsgestaltung gesehen [18, 27].

Vor diesem Hintergrund umfasst die vorliegende Untersuchung einerseits eine quantitative Bestandsaufnahme in Bezug auf die psychische Gesundheit und das Wohlbefinden der Studierenden im Zuge des Wintersemesters 2020/2021. Darüber hinaus werden die erlebten Studienbedingungen mit besonderem Blick auf das durch die Pandemie verursachte Online-Studium als wichtige Faktoren in Bezug auf die psychische Gesundheit und das Wohlbefinden im qualitativen Design untersucht.

Die Zielgruppe dieser Studie sind Studierende der Hochschule für Gesundheit in Bochum, die im Wintersemester 2020/21 studierten. An der Hochschule werden insgesamt elf gesundheitswissenschaftliche Bachelor- und Masterstudiengänge angeboten. Im Wintersemester 2020/2021 waren 1755 Studierende an der Hochschule immatrikuliert. Davon waren 1467 (84 %) weiblich. Zum Zeitpunkt der Erhebung, im März 2021, dem sog. zweiten Lockdown, findet das Studium bereits seit fast einem Jahr weitestgehend online statt.

Im Folgenden soll daher der Frage nachgegangen werden, wie im Wintersemester 2020/2021 die psychische Gesundheit und das Wohlbefinden Studierender der Hochschule für Gesundheit in Bochum ausgeprägt waren.

## Methode

Um die beschriebenen Fragestellungen zu beantworten, wurde ein quantitativer sowie ein qualitativer Ansatz gewählt. Die beiden Untersuchungen werden nachfolgend jeweils voneinander getrennt dargestellt.

### Quantitative Erhebung des psychischen Gesundheitszustands der Studierenden mittels Fragebogen

Zur Untersuchung der psychischen Gesundheit der Studierenden wurde ein Online-Fragebogen entwickelt. Ein Link zu diesem wurde im März 2021 über einen E‑Mail-Verteiler der Hochschule für Gesundheit an alle Studierenden versendet. Dieser war vier Wochen aktiv. Für die Teilnahme wurde ein Mindestalter von 18 Jahren, die Immatrikulation an der Hochschule für Gesundheit im Wintersemester 2020/2021 sowie die Zustimmung der Einverständniserklärung vorausgesetzt.

Der Fragebogen enthielt Fragen zu demografischen Angaben (Alter, Geschlecht, Studiengang, Fachsemester) sowie zu den Merkmalen Wohlbefinden und psychischer Gesundheit. Dazu wurden der WHO Five Well-Being Index, kurz WHO-5 [29] sowie die deutsche Version des Patient Health Questionnaire (PHQ) [12] eingesetzt.

Der WHO‑5 wurde zur Ermittlung des Wohlbefindens gewählt. Er wurde als Screeninginstrument von der WHO entwickelt, um das Wohlbefinden zu erfassen und wird in der Leitlinie zur Erfassung unipolarer Depressionen empfohlen [7]. Das Wohlbefinden wurde durch einen Gesamtscore von 0 (geringstes Wohlbefinden) bis 25 (höchstes Wohlbefinden) ermittelt. Der Gesamtscore ergab sich durch die Auftretenshäufigkeit von fünf Symptomen des Wohlbefindens innerhalb der letzten zwei Wochen auf einer 6‑stufigen Skala 0 = „zu keinem Zeitpunkt“, 1 = „ab und zu“, 2 = „etwas weniger als die Hälfte der Zeit“, 3 = „etwas mehr als die Hälfte der Zeit“, 4 = „meistens“, 5 = „die ganze Zeit“. Die WHO [29] empfiehlt zudem ab einem Cut-off-Wert von 12 eine weiterführende Testung auf eine Depression.

Zur Untersuchung der psychischen Gesundheit wurde die Kurzform des PHQ [19] zur Ermittlung depressiver Störungen in der deutschen Version (PHQ-D) gewählt [12]. Da er aus neun Items besteht, wird er als PHQ‑9 abgekürzt. Er wurde auf Grundlage der diagnostischen Kriterien des Diagnostic and Statistical Manual of Mental Disorders (DSM-IV) [1] als Screeninginstrument für die Primärmedizin entwickelt und ermöglicht eine dimensionale Auswertung von depressiven Störungen und die Bestimmung des Schweregrads (depressive Syndrome, DS oder major depression syndrome, MDS). Bei der Validierung der deutschen Version konnte mit einer Sensitivität von 95 % und einer Spezifität von 86 % eine ausgezeichnete Kriteriumsvalidität bei der Diagnose des MDS gezeigt werden [12]. Auf einer 4‑stufigen Antwortskala von 1 = „überhaupt nicht“, 2 = „an einzelnen Tagen“, 3 = „an mehr als der Hälfte der Tage“ bis 4 = „beinahe jeden Tag“ sollte, bezogen auf die letzten zwei Wochen, das Auftreten von neun verschiedenen depressiven Symptomen des DSM-IV eingeschätzt werden. Ein depressives Syndrom lag demnach vor, wenn die Hauptsymptome „wenig Interesse oder Freude an deinen Tätigkeiten“ sowie „Niedergeschlagenheit, Schwermut oder Hoffnungslosigkeit“ an mindestens „mehr als der Hälfte der Tage“ in den letzten zwei Wochen aufgetreten sind. Traten daneben noch mindestens drei weitere Symptome an „mehr als der Hälfte der Tage“ auf, liegt ein MDS vor. Dem Auftreten von Suizidgedanken wurde eine besondere Bedeutung zugemessen, indem es bereits ab der Ausprägung 2 = „an einzelnen Tagen“ berücksichtigt wurde. Das Instrument wurde gewählt, da repräsentative Vergleichsdaten zur Prävalenz des DS bei Studierenden [12] vorliegen und es in nationalen Bevölkerungsstudien Anwendung findet [6, 16]. Außerdem wird es als Instrument für die Früherkennung depressiver Störungen in der S3-Leitlinie empfohlen [7].

Die Auswertung der Daten erfolgte im April 2021 mittels des Statistikprogramms SPSS (IBM Corp. Released 2017. IBM SPSS Statistics for Windows, Version 25.0. Armonk, NY, USA). Für den WHO‑5 wurden Gesamtscores und entsprechende Mittelwerte gebildet. Außerdem wurde eine Gruppeneinteilung entsprechend des Cut-off-Wertes vorgenommen. Zur Auswertung des PHQ‑9 wurden individuelle Profile erstellt, die eine Einteilung in die Kategorien „Kein Hinweis auf ein depressives Syndrom“, „depressive syndrome“ oder „major depression syndrome“ ermöglichen.

### Qualitative Befragung der Studierenden zu ihrem Erleben der Studienbedingungen mittels Interviews

Zur Untersuchung der neuen Studienbedingungen im Wintersemester 2020/2021, welches weitestgehend online unterrichtet wurde, wurden Teilnehmende für eine Interviewstudie per E‑Mail gesucht. Im März 2021 wurde eine E‑Mail an alle Studierenden der Hochschule geschickt. Auf die Anfrage meldeten sich insgesamt acht Studierende, davon sieben weiblich und einer männlich. Es wurden alle eingeschlossen, da sie mindestens 18 Jahre alt waren, im Wintersemester 2020/2021 an der Hochschule studierten und der Einverständniserklärung zustimmten. Die Interviews erfolgten in der Zeit vom 12.03.2021 bis zum 26.03.2021 über die Online-Videotelefonieplattform Zoom und wurden im Rahmen einer Masterarbeit von Studierenden durchgeführt. Die Teilnehmenden studierten in allen drei Departments der Hochschule durchschnittlich im 4. (Range: 2. bis 9.) Semester. Die Dauer der Interviews betrug im Durchschnitt 28 (Range: 14–39) min. Um das Erleben des Online-Studiums aus Sicht der Studierenden in einem möglichst offen gestalteten Interview zu erheben, wurde der Ansatz eines problemzentrierten und leitfadengestützten Interviews gewählt [22]. Die offene Sondierungsfrage „Erzähl mir doch mal, wie dein Studium seit Corona ablief …“ wurde zu Beginn des Interviews gestellt, um den Einstieg für ein offenes Interview zu schaffen, indem der*die Interviewte thematische Schwerpunkte setzen konnte. Themen des Leitfadens waren der Studienalltag, das Selbststudium, allgemeine gesundheitliche Veränderungen seit der Pandemie, Nachteile und Stressoren sowie Vorteile und Chancen des Online-Studiums. Daraus abgeleitet wurden Aufrechterhaltungs- und Steuerungsfragen, die bei Bedarf gestellt wurden. Aufgrund der offen formulierten Sondierungs- und Einstiegsfragen konnten die Interviews zu einem großen Teil von den Interviewten thematisch gelenkt werden. Nach der Durchführung wurden die Interviews umgehend transkribiert. Anschließend wurde eine kategorienbasierte Datenauswertung in Anlehnung an die qualitative Inhaltsanalyse nach Kuckartz [20] durchgeführt. Deduktiv wurden dabei sechs Hauptkategorien festgelegt, anhand derer die Kodierung des Materials vorgenommen wurde. Die Kodierung erfolgte konsensuell, wobei zwei Forscherinnen in einem ersten Schritt unabhängig kodierten, um die Kodierungen in einem zweiten Schritt gemeinsam zu diskutieren und zu prüfen. In einem weiteren Schritt wurden gemeinsam induktiv Unterkategorien gebildet, um eine größere Detailtiefe zu erreichen. Die Kategorien lassen sich dem Ergebnisteil entnehmen.

## Ergebnisse

### Psychischer Gesundheitszustand der Studierenden

Insgesamt nahmen 412 Studierende an der Online-Umfrage teil (23,5 %). Davon waren 86,2 % weiblich und 11,2 % männlich. Das durchschnittliche Alter betrug 25,6 Jahre. 90,3 % füllten die Umfrage vollständig aus.

**Abb. 1:**
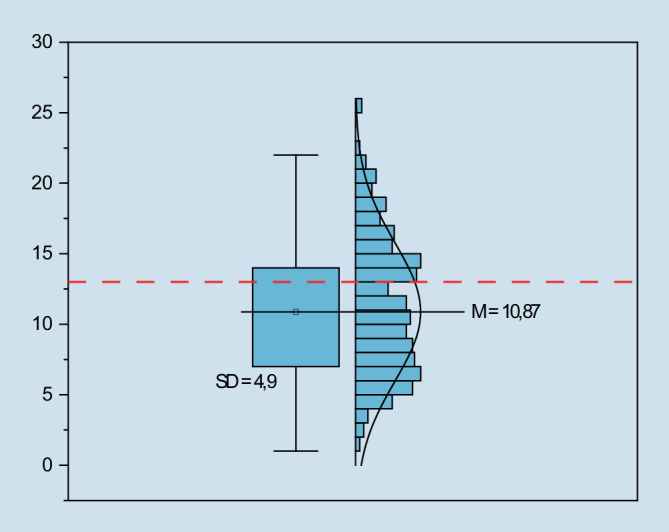
WHO-5-Gesamtscore (WHO Five Well-Being Index) aus der Befragung 2021 (Range 0–25)

Der WHO‑5 wurde von 394 Befragten vollständig ausgefüllt. Im Mittel erreichten die Studierenden einen Wert von 10,87 (Range: 0–25, SD: 4,90). Einen Wert von < 13 und damit ein besonders niedriges Wohlbefinden zeigte sich bei 68 % der Befragten. In Abb. 1 werden die Ergebnisse des WHO-5-Fragebogens zusammenfassend dargestellt.

**Abb. 2:**
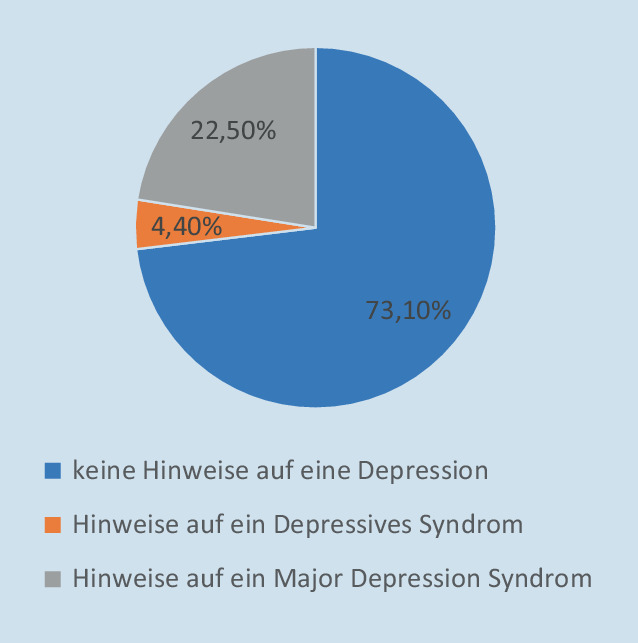
Ergebnisse PHQ‑9 („patient health questionnaire“) 2021

Der PHQ-9-Fragebogen wurde von 383 Teilnehmenden vollständig ausgefüllt. 73,1 % zeigten keine Hinweise auf eine Depression. Von den übrigen 26,9 % der Befragten erfüllte nur ein geringer Teil von 4,4 % die Kriterien für ein DS. Die restlichen 22,5 % ließen sich dem MDS zuordnen. In Abb. 2 werden die Ergebnisse des PHQ-9-Fragebogens zusammenfassend dargestellt.

### Erleben der Studienbedingungen aus Perspektive der Studierenden

#### Negative Aspekte

Die Studierenden berichteten hauptsächlich von negativen Aspekten des Online-Studiums. Negative Aspekte wurden von den Studierenden zu einem großen Teil auf eine veränderte Kommunikationssituation zurückgeführt. Darunter falle sowohl der fehlende Austausch mit Kommiliton*innen, als auch mit Lehrenden. Dabei wurden sowohl der fehlende Austausch über Studieninhalte und organisatorische Fragen als auch ein persönlicher Austausch über das Studium hinaus als ein maßgeblich negativer Faktor von insgesamt sieben Befragten genannt. Eine Interviewpartnerin beschreibt ihre Empfindungen, die sie aufgrund des fehlenden Austauschs hat als „sehr anonym und der Austausch auf sämtlichen Ebenen […] das fehlt komplett.“ (I. 1, Z. 191–194). Als ein Grund für die nicht ausreichende Kommunikation wurde von vier Studierenden betont, dass die digitale Kommunikationssituation nicht ganzheitlich sei und Hürden für einen Austausch darstellen würden. Darüber hinaus wurden negative Auswirkungen der digitalen Home-Office-Situation wie ungünstige räumliche Bedingungen zuhause und fehlende Trennung von Arbeit und Freizeit, da „die Uni jetzt [bei ihnen] zuhause“ sei (I. 4, Z. 267–279) von Studierenden berichtet. Dadurch käme es häufiger zu Ablenkungen und eine Konzentration auf das Studium gestalte sich schwierig. Die Konzentrationsfähigkeit werde zudem durch die längere Bildschirmzeit beansprucht. Für das Studieren ergäben sich weiterhin für die befragten Studierenden Probleme durch wegfallende Strukturen und einen Mehraufwand bei der Selbstorganisation. Es würde sich von den Lehrenden mehr Input und Unterstützung gewünscht. Neben den Problemen im Studium hätten Studierende auch im Privaten mit den Auswirkungen der Pandemie zu kämpfen. Der fehlende private Ausgleich und soziale Kontakte konnte neben der Unsicherheit, die sich aus den Entwicklungen der Pandemie ergeben, als Hauptsorgen der Studierenden neben dem Studium herausgearbeitet werden.

#### Positive Aspekte

Vorteile, die sich für die Studierenden aus dem Online-Studium ergäben, waren wegfallende Fahrtwege, eine flexiblere Arbeitsgestaltung und Entlastungen in den Prüfungen durch Freiversuche und Open-book-Klausuren. Wobei eine Interviewpartnerin dies als „keinen mega Pluspunkt“ und mit den Worten „nice to have“ beschreibt (I. 1, Z. 176–179).

#### Psychische und physische Gesundheit

Sieben der acht Befragten schilderten Ermüdungserscheinungen, Schlafstörungen, psychische Instabilität oder Gefühle wie Traurigkeit, Wut, Angst und Stress. Diese führten die Befragten zum einen auf das Wegbrechen von privatem und sozialem Ausgleich und zum anderen auf die fehlende Motivation für das Studium zurück. Bezogen auf das Studium fallen Schlagworte wie „zermürbend“ (I. 6, Z. 12–13), „schlaucht“, „ausgelaugt“ (I. 3, Z. 28–32), „sehr sehr anstrengend“ (I. 6, Z. 22–23) sowie „ultra stressig und belastend“ (I. 3, Z. 226). Dabei kann ein Zusammenspiel aus privatem und studienbedingten Stress vermutet werden. Auch auf Sport und Ernährung nahm die Pandemie im beobachteten Zeitraum bei den Studierenden Einfluss. Hierbei konnte dazugewonnene Zeit zu verbesserten Koch‑, Ess- und Sportgewohnheiten beitragen. Allerdings schien das Online-Studium bei den Studierenden insgesamt zu weniger Bewegung und mehr Rücken‑, Kopf- und Augenschmerzen zu führen.

#### Coping-Strategien

Im Umgang mit Belastungen im studentischen Alltag wurden Ortswechsel als hilfreich beschrieben, um die Lernsituation zu verbessern. Auch der Austausch mit Kommiliton*innen und sozialen Kontakten außerhalb der Hochschule wurde als entlastend empfunden, insofern dieser möglich war. Ein digitaler Austausch wurde, wie oben bereits erwähnt, nicht als ausreichend empfunden. Profitiert würde von einer telefonischen Inanspruchnahme der psychosozialen Beratung der Hochschule. Auch sportliche Aktivitäten und Spaziergänge würden in der Pandemie wertgeschätzt.

#### Wünsche

Konkrete Wünsche wurden an Dozierende insbesondere bei der Lehrgestaltung gerichtet. Zum Beispiel in einer strukturierteren Darbietung und Nutzung digitaler Lehr- und Lernmöglichkeiten neben einem breiteren Angebot sich auszutauschen und Fragen zu klären. Aber auch der Institution Hochschule wurde eine wichtige Rolle bei der Gesunderhaltung von den Studierenden zugesprochen. Studierende wünschten sich Angebote an der Hochschule zum Thema Gesundheit und zur persönlichen Gesunderhaltung im Alltag. Dabei wurden konkret die Bereiche Resilienz, Motivation, Ernährung, Stressmanagement und positives Gesundheitsverhalten genannt.

## Diskussion

### Psychischer Gesundheitszustand der Studierenden

Die Untersuchungsergebnisse zum Wohlbefinden und zur psychischen Gesundheit Studierender der Hochschule für Gesundheit in Bochum während des Untersuchungszeitraums zeigen ein unterdurchschnittlich ausgeprägtes Wohlbefinden bei überdurchschnittlich hohem Anteil an Studierenden mit dem Verdacht auf das Vorliegen eines depressiven Syndroms. Die Ausprägung des Wohlbefindens lag bei den Befragten mit einem Mittelwert von 10,89 Punkten bereits unterhalb des Cut-off-Wertes von 13 Punkten, unterhalb dessen Hinweise auf eine Depression vorliegen. Der prozentuale Anteil von Teilnehmenden, die einen Punktwert < 13 erreichten, lag bei 68 (vgl. Abb. 2). Repräsentative Vergleichsdaten aus der deutschen Allgemeinbevölkerung liegen von 2018 vor der Pandemie und 2020 während der Pandemie vor [13]. Es konnten hier bei 34,5 % (2018) und 36,7 % (2020) der Befragten Hinweise für das Vorliegen einer Depression ermittelt werden. Im Vergleich zu den Daten der Allgemeinbevölkerung sowohl vor als auch während der COVID-19-Pandemie wird ein überdurchschnittlich hoher Anteil an Studierenden mit einem auffällig geringen Wohlbefinden deutlich. Dies zeichnet sich ebenso bei den Werten zur psychischen Gesundheit ab. Bei dieser Stichprobe zeigten nach den Kriterien des PHQ‑9 mehr als ein Viertel der Studierenden (26,9 %) Hinweise für das Vorliegen einer Depression, wovon bei 22,5 % entsprechend der Interpretation des Summenwertes eine besonders schwere Form der Depression, die sog. „major depression“ vermutet werden kann. Im Vergleich dazu wurden in einer deutschlandweiten Umfrage 2017 bei 15,6 % der befragten Studierenden mit dem PHQ‑9 Hinweise für das Vorliegen einer Depression erhoben [16]. Diese Daten liegen bereits über dem nationalen Durchschnitt von 9,4 % [6]. Insgesamt wird deutlich, dass befragte Studierende während des Wintersemesters 2020/2021 im Vergleich zu Untersuchungen in der Allgemeinbevölkerung und mit Studierenden ein vergleichsweise gering ausgeprägtes Wohlbefinden bei erhöhtem Verdacht auf das Vorliegen von einer Depression aufwiesen.

Um das Erleben der Studierenden während des zweiten Lockdowns und des Online-Studiums besser abbilden zu können, wurde für eine weiterführende Untersuchung ein qualitatives Design gewählt.

### Erleben der Studienbedingungen aus Perspektive der Studierenden

Die Analyse der Interviews ergab ein hauptsächlich negativ geprägtes Erleben des Online-Studiums seitens der Studierenden. In der vorliegenden Untersuchung schien die fehlende bzw. veränderte Kommunikationssituation den größten Einfluss zu haben. Studierende wurden sowohl durch das Wegfallen von privatem und studienbezogenem Austausch als auch durch Veränderungen der Kommunikationssituation vor eine Vielzahl von Herausforderungen gestellt. Dazu zählten Gefühle wie Isolation, Einsamkeit und Überforderung, aber auch inhaltliche und organisatorische Probleme im Studium. Auch in Bezug auf diese genannten Aspekte mit Bezug zur Kommunikationssituation während der Pandemie stimmen die Aussagen der Studierenden der HS Gesundheit mit den Ergebnissen ähnlicher Untersuchungen an anderen Hochschulen überein [2, 15, 33]. Studierende mit psychischen Vorerkrankungen berichteten von einer subjektiven Verschlechterung psychischer Belastungen, die mit der Sorge einhergingen, ihr Studium nicht abschließen zu können. In den Schilderungen spielten auch individuelle Belastungen wie elterliche Pflichten neben dem Studium eine Rolle. Auch das Online-Studium erzeugte Stress, der aufgrund von fehlendem privatem Ausgleich von den Studierenden als belastend wahrgenommen wurde. Dies deckt sich mit den Ergebnissen einer Untersuchung von Hahn, Kuhlee und Porsch aus dem Jahr 2021 [14], in der sie die Konsequenzen der Verlagerung der Lehrangebote in den digitalen Raum bei Lehramtsstudierenden untersuchten. Als Entlastungen empfanden die Studierenden im Online-Studium Freiversuche, „open book“ und wegfallende Fahrtwege. Die negativen Aspekte standen jedoch im Fokus der Berichte. Dies deckt sich mit Forschungsergebnissen aus anderen Hochschulen. Auch hier empfanden Studierende die Online-Lehre nicht als ausreichenden Ersatz für die Präsenzlehre [5]. Isolation und der fehlende Kontakt zu Studierenden fiel auch dabei besonders ins Gewicht [5, 17, 27].

Alle Befragten schilderten, dass sie während des Online-Studiums Symptome psychischer Belastungen erlebt haben. Die körperliche Gesundheit stand nicht im Fokus der Berichte und wurde sowohl positiv als auch negativ berichtet.

Es lässt sich festhalten, dass Studierende sich sowohl von Seiten der Dozierenden als auch von der Hochschule mehr Unterstützung während des pandemiebedingten Online-Studiums gewünscht hätten. Durch die Äußerung des konkreten Wunschs Studierender nach Unterstützung der Hochschule wird das Potenzial der Hochschule auch aus Perspektive der Studierenden für ein gesundheitsförderliches Setting deutlich.

## Methodische Reflexion

Abschließend soll hier noch einmal deutlich festgehalten werden, dass die Befragungsergebnisse aus der quantitativen Untersuchung nicht als psychiatrische oder psychotherapeutische Diagnosestellung zu verstehen sind. Die Dynamik der COVID-19-Pandemie sowie der damit einhergehenden politischen Maßnahmen schränkt die Aussagekraft der Ergebnisse hier insofern ein, als dass die Ergebnisse der Querschnittuntersuchung als punktuell betrachtet werden sollten. Für den untersuchten Zeitraum können die gewonnenen Daten jedoch einen ersten Eindruck der psychischen Gesundheit von Studierenden an der Hochschule für Gesundheit liefern, den es kontinuierlich zu prüfen gilt.

Mit Hilfe des qualitativen Untersuchungsstrangs konnte ein Einblick in das Erleben des Online-Studiums aus Perspektive der Studierenden ermöglicht werden, die mit Hilfe eines offen gestalteten Interviewleitfadens und von Studierenden durchgeführt wurden. Hier ist zu berücksichtigen, dass es sich um individuelle Erfahrungen von Studierenden der Hochschule für Gesundheit handelt und eine Übertragbarkeit auf andere Hochschulen nicht per se möglich ist. Untersuchungsergebnisse anderer Hochschulen deuten jedoch auf ähnliche Erfahrungen hin [2]. Ein größeres Sampling wäre wünschenswert gewesen, um einen umfassenderen Einblick in das Erleben der Studierenden gewinnen zu können. An dieser Stelle wäre möglicherweise die Erweiterung der Akquise auf weitere Kommunikationskanäle gewinnbringend gewesen. Dennoch konnten Erfahrungen von Studierenden aus allen Departments der Hochschule und aus verschiedenen Semestern abgebildet werden. Aufgrund der vielfältigen und neuartigen Auswirkungen der COVID-19-Pandemie ist ein multikausaler und individueller Einfluss u. a. auf die psychische Gesundheit zu vermuten [24].

Die beiden vorgestellten Untersuchungen zeigen zunächst die Bedeutung eines regelmäßigen Gesundheitsmonitorings an Hochschulen sowie den regelmäßigen Bedarf an Evaluation bzw. Überprüfung entsprechender Befunde auf. Damit sind vor allem die Hochschulen im Sinne des Settingansatzes gefragt, die Gesundheit ihrer Studierenden im Blick zu haben. Sicher stammen die hier vorgelegten Befunde aus der Betrachtung einer Extremsituation. Gleichzeitig zeigen sie aber auch, wie umfangreiche Informationen sich durch Befragung und Dialog mit Studierenden einerseits für die praktische Gesundheitsförderung sowie andererseits für die Wissenschaft gewinnen lassen. Die gewonnenen Erkenntnisse zu den Herausforderungen Studierender im Online-Studium können vor dem Hintergrund zunehmender Home-Office-Arbeit und der Digitalisierung im Allgemeinen auch in anderen Kontexten weiter untersucht werden, um Problemen frühzeitig gezielt entgegen wirken zu können.

## Fazit für die Praxis

- Studierende sind vulnerabel für psychische Belastungen.
- Viele der möglichen Bedingungen, die einen negativen als auch positiven Einfluss auf Studierenden und ihre psychische Gesundheit haben, können durch die Hochschule gestaltet werden. Ihr kommt eine wichtige Rolle als Akteur hinsichtlich der psychischen und physischen Gesundheit der Studierenden und Mitarbeitenden zu.
- Es müssen Strukturen geschaffen werden, die den Studierenden im Sinne einer Gesundheitsförderungskultur einen gesundheitsförderlichen Lebensstil im Setting Hochschule und darüber hinaus ins Berufsleben ermöglichen. Sie müssen darin befähigt werden, insbesondere ihre psychische Gesundheit zu schützen und zu fördern. Hierfür eignet sich ein partizipativ gestaltetes studentisches Gesundheitsmanagement.
- Eine regelmäßige standardisierte Evaluierung muss dabei gewährleistet werden, um Maßnahmen aktuell, problem- und zielgruppenorientiert auszurichten.
